# Efficacy of ethyl chloride topical anesthesia application on pain perception during intra-oral injections in children compared to benzocaine gel—a single-blind randomized controlled trial

**DOI:** 10.3389/fdmed.2026.1925109

**Published:** 2026-08-19

**Authors:** Nagah Abdelrahman, Manal Al Halabi, Mawlood Kowash, Iyad Hussein, Anas Salami

**Affiliations:** Hamdan Bin Mohammed College of Dental Medicine, Mohammed Bin Rashid University of Medicine and Health Sciences, Dubai Health, Dubai, United Arab Emirates

**Keywords:** benzocaine, cryoanesthesia, ethyl chloride, local anesthesia, pain perception, pediatric dentistry, topical anesthesia

## Abstract

**Background:**

Pain management during local anesthesia administration is a critical factor in pediatric dentistry. This assessor-blind, parallel-group, superiority randomized controlled trial (ClinicalTrials.gov: NCT06011005) aimed to determine whether ethyl chloride (EC) spray applied via a cotton pellet provides superior pain reduction compared to 20% benzocaine (BC) gel during intra-oral local anesthetic injections in children.

**Methods:**

Fifty-seven healthy children aged 7–10 years requiring routine dental local anesthesia were randomized to Group A (20% BC gel, *n* = 29) or Group B (EC spray via cotton pellet, *n* = 28). The primary outcome was physiological stress measured by change in heart rate (HR). Secondary outcomes included self-reported pain (Faces Pain Scale, FPS) and observed behavioral response (Sound-Eye-Motor scale, SEM). The outcomes assessor was blinded to the topical agent used. Between-group differences in change scores were analyzed using the Mann–Whitney *U* test with Hodges–Lehmann median differences and bootstrap 95% confidence intervals.

**Results:**

Both groups demonstrated slight increases in HR following the injection; however, the magnitude of change was not significantly different between the groups (BC: +4.03 bpm vs. EC: +2.14 bpm; Mann–Whitney *U p* = 0.310; 95% CI of mean difference: −1.53 to 5.35). Additionally, there were no statistically significant differences between the groups regarding self-reported pain levels (FPS change: BC +1.31 vs. EC +1.25; *p* = 0.546; 95% CI: −1.03 to 1.08) or behavioral responses (SEM change: BC +0.10 vs. EC +0.11; *p* = 0.971; 95% CI: −0.22 to 0.21).

**Conclusion:**

The superiority trial did not reveal a statistically significant difference between the use of ethyl chloride spray and 20% benzocaine gel across any of the measured outcomes. Both anesthetic agents produced comparable physiological responses, self-reported experiences, and behavioral reactions during intra-oral local anesthetic injections in cooperative children aged 7–10 years.

## 1 Introduction

Local anesthesia is a fundamental component of pediatric dental practice. However, the injection itself is often the most anxiety-provoking and painful aspect of the dental visit for children (1, 2). To mitigate injection pain, topical anesthetic agents are routinely applied to the oral mucosa prior to needle insertion. Benzocaine gel (20%) is the most widely used topical anesthetic in pediatric dentistry, providing surface anesthesia through reversible blockade of sodium channels in peripheral nerve endings (3, 4).

Despite its widespread use, benzocaine gel has notable limitations. Its onset of action requires 1–3 min of mucosal contact, and its efficacy is confined to the superficial 2–3 mm of tissue (3). Furthermore, rare but serious adverse effects, including methemoglobinemia and contact allergic reactions, have been documented (5, 6). These limitations have prompted the exploration of alternative pre-injection anesthetic strategies.

Cryoanesthesia—the application of cold to induce local numbness—represents a fundamentally different mechanism of topical pain control. Cooling the tissue to approximately 4 °C slows nerve conduction velocity and raises the pain threshold by reducing the activity of nociceptive C-fibers and Aδ-fibers (5, 7). Ethyl chloride (EC) is a volatile liquid that evaporates rapidly at body temperature, producing a transient freezing effect on the applied surface (8). It has been used extensively in medicine for procedural pain (e.g., venipuncture, intravenous cannulation) with a well-established safety profile on intact skin (9–11). Historically, ethyl chloride was employed as a topical anesthetic for dental extractions in children in the mid-20th century (12, 13), but its intra-oral use fell out of favor with the advent of modern topical gels.

The application of cryoanesthesia to the oral mucosa, however, has received comparatively limited investigation. Several studies have examined ice or refrigerant sprays as pre-injection topical agents in adults (8, 14–17), but evidence in pediatric populations has historically been sparse. A recent scoping review by Sanglard et al. analyzed 52 randomized controlled trials of topical anesthetic methods in children and identified cooling-based approaches (including ice) among the most effective strategies alongside lidocaine and benzocaine (18). Additionally, Ninawe and Anija reported that ice application produced comparable pain scores to benzocaine gel in children receiving local anesthesia via a computer-controlled delivery device (19). Furthermore, Abbasi et al. (20) recently compared direct ethyl chloride spray against 5% lidocaine gel for buccal infiltration anesthesia in adults and reported comparable pain scores between the two methods (20, 21).

Despite these emerging data, no published trial has specifically evaluated the indirect application of ethyl chloride spray via a cotton pellet as a pre-injection topical anesthetic for intra-oral use in children. This indirect technique of spraying EC onto a cotton pellet until frost forms, then applying the frosted pellet to the mucosa was designed to avoid the risks of direct intra-oral spraying (aspiration, uncontrolled tissue exposure) while harnessing the rapid-onset cryoanesthetic effect.

The aim of this study was to determine whether this novel indirect EC application technique provides superior reduction in injection-related pain and physiological stress compared to conventional 20% benzocaine gel in children aged 7–10 years undergoing routine intra-oral local anesthesia infiltration.

## 2 Materials and methods

### 2.1 Study design and settings

**Figure 1:**
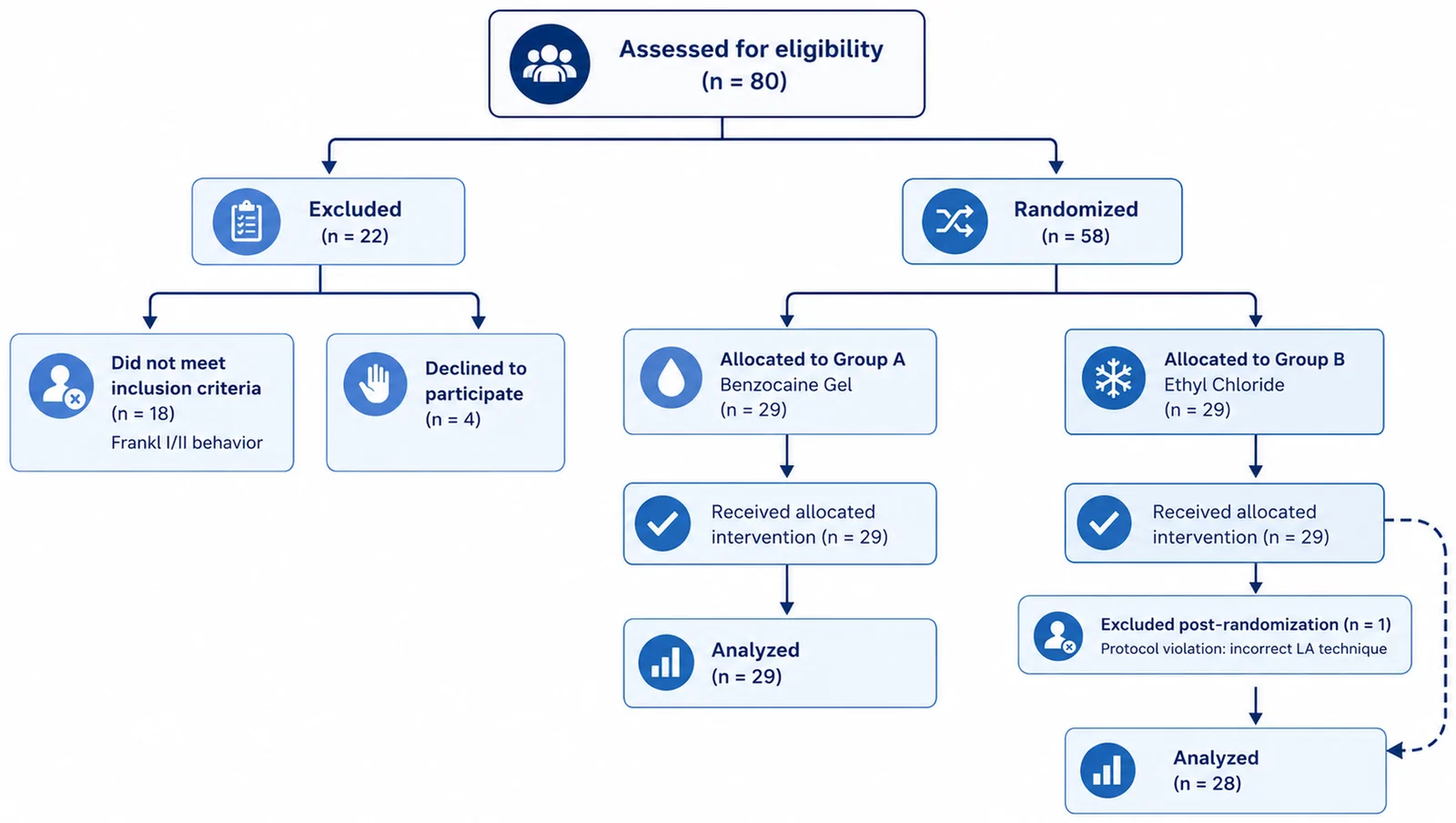
CONSORT-2025 flow diagram of participant recruitment, allocation, and analysis.

This study was a single-center, parallel-group, assessor-blind, superiority randomized controlled trial. It was prospectively registered at ClinicalTrials.gov (NCT06011005) and approved by the Institutional Review Board of Mohammed Bin Rashid University of Medicine and Health Sciences (MBRU-IRB-2022-048). The trial was conducted in accordance with the Declaration of Helsinki and is reported following the CONSORT 2025 guidelines (22, 23) (Figure 1).

The registered protocol specified the following parameters that were subsequently amended prior to data collection: (a) the Visual Analog Scale (VAS) was removed as an outcome measure upon recommendation from the thesis examination committee, as the Faces Pain Scale (FPS) was considered more developmentally appropriate and reliable for this pediatric age group, rendering the VAS redundant; (b) the EC spray distance was refined from 10 to 5 cm based on pilot testing (*n* = 6) which demonstrated that 5 cm produced more consistent frost formation on the cotton pellet; (c) the local anesthetic delivery system was changed from a non-aspirating syringe (1.8 mL) to a standard aspirating syringe (0.5 mL) to comply with institutional clinical protocols; and (d) the group coding was reversed from the registry (registry: Group A = EC, Group B = BC; executed: Group A = BC, Group B = EC) to match the chronological order of patient enrollment. These amendments were approved by the supervising faculty and did not alter the fundamental research question or primary outcome. No changes to the trial design occurred after commencement of participant enrollment.

### 2.2 Sample size calculation

The sample size was calculated based on anticipated differences in child pain scores between groups. Using estimates from a related pediatric topical anesthesia trial (assuming a 20% relative difference in pain scores, analogous to a mean difference of ∼20 on a 100-point VAS, SD ∼12), a minimum of 21 children per group was estimated to achieve 80% power at a 5% significance level for detecting a difference in pain scores. To account for potential dropouts, we aimed to recruit at least 30 children per group (60 total). Ultimately, 57 children were enrolled and completed the trial, which provided adequate power for the primary outcome comparisons. *Post hoc*, the observed between-group difference in heart rate change (effect size *d* = 0.82) confirmed that the study was adequately powered for the physiological outcome; however, we acknowledge that the original power calculation was based on a VAS scale rather than the FPS or HR scales used, which is a limitation.

### 2.3 Participants and eligibility criteria

Children aged 7–10 years attending the pediatric dental clinic for routine dental treatment requiring local anesthesia were assessed for eligibility.

Inclusion criteria were:

- healthy children classified as ASA I who required routine dental treatment involving a maxillary buccal infiltration injection (anterior, middle, or posterior region);
- no prior exposure to dental local anesthesia or ethyl chloride;
- cooperative behavior categorized as Frankl III (positive) or IV (definitely positive);
- no current use of analgesics or medications affecting pain perception; and
- no history of allergic reactions to local anesthetics or ethyl chloride.

Exclusion criteria included:

- uncooperative behavior (Frankl I or II) or history of needle phobia;
- presence of acute dental infection or abscess at the injection site;
- systemic diseases or cognitive impairments affecting pain perception or communication; and
- previous traumatic dental experiences.

Written informed consent was obtained from parents or legal guardians along with verbal child assent before randomization, including specific consent for the publication of anonymized clinical photographs. The investigators monitored the study for any adverse events; no adverse effects from either topical anesthetic method were observed.

### 2.4 Pilot testing and calibration

The entire methodology was pilot-tested on 6 children (3 per group) who were not part of the main study, to validate the feasibility and refine the protocols. The operator was calibrated with an experienced pediatric dentist to ensure consistency in both topical anesthetic application and local injection techniques. The outcome evaluator was similarly calibrated in the use of the SEM behavioral scale by scoring sample videos of children undergoing injections, achieving 100% intra-rater agreement over two rounds of scoring.

### 2.5 Randomization and allocation concealment

Participants were randomly assigned (1:1) to Group A (Benzocaine gel) or Group B (Ethyl Chloride spray) using a computer-generated randomization sequence. Allocation concealment was maintained using sequentially numbered, opaque, sealed envelopes prepared by an independent researcher not involved in patient recruitment or treatment.

### 2.6 Blinding

This trial employed an assessor-blind design. The operator (AA) opened the allocation envelope and administered the assigned topical anesthetic; therefore, the operator was not blinded. Due to the inherent thermal and tactile differences between the −20 °C EC cotton pellet and the room-temperature benzocaine gel, it was physically impossible to blind the child participants to the intervention received, and children were not explicitly informed which agent was being used; Tell–Show–Do behavior guidance and protective eyewear were employed to standardize the experience. However, the outcomes assessor (NA) was strictly blinded to group allocation. To maintain this blinding, the assessor was not present in the operatory during the application of the topical anesthetic. Once the topical agent had been applied, removed, and the child's mouth rinsed to eliminate any residual visual or olfactory cues, the assessor entered the room to observe the injection and record the physiological and behavioral outcomes.

### 2.7 Interventions

All interventions were conducted by a single calibrated operator (AA). In Group A, a 20% benzocaine gel (Topical Anesthetic Gel, Hurricaine®, Beutlich Pharmaceuticals) was meticulously applied to the dried oral mucosa at the designated injection site using a cotton applicator, with a saliva ejector in place to aspirate excess gel. The application duration ranged from 2 to 3 min. A minimum application time of 2 min was maintained in cases where isolation was not optimal, or if the child experienced discomfort from the taste or began swallowing the gel. In all other instances, efforts were made to ensure that the gel was applied for a full 3 min for each participant.

**Figure 2:**
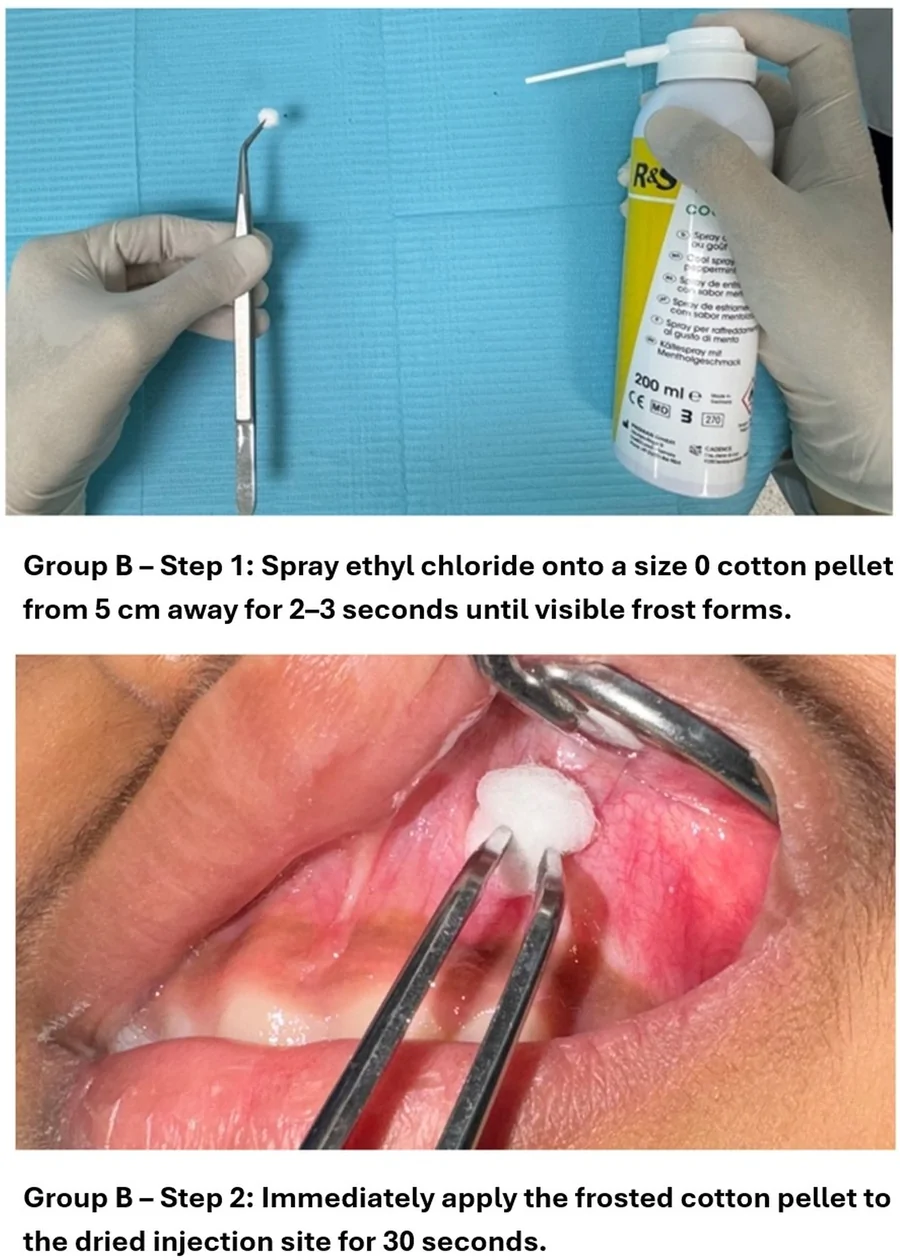
Steps of indirect ethyl chloride spray (Group B) application technique via frosted cotton pellet on buccal mucosa.

In Group B, ethyl chloride spray (Kryogen®, R&S, Italy; cooling to approx. −20 °C) was applied indirectly. The operator sprayed a size 0 cotton pellet with ethyl chloride for 2–3 s from a distance of 5 cm until visible frost formed on the pellet surface. The frosted cotton pellet was then immediately placed against the dried oral mucosa at the injection site for 30 s (A dental nurse utilized a timer to ensure the operator was informed about the appropriate application duration). Care was taken to avoid incidental contact with adjacent tooth crowns to prevent transient pulpal sensitivity (24, 25) (Figure 2).

Following topical application in both groups, the operator was strictly calibrated to avoid incidental contact between the chilled pellet and the adjacent tooth crown, preventing any pulpal thermal stimulation. To standardize the injection procedure, the same experienced operator (a pediatric dentist) administered the subsequent local anesthetic injection to all participants. A 27-gauge short needle attached to a standard aspirating syringe was used to deliver ∼0.5 mL of 2% lidocaine with 1:100000 epinephrine by a standard buccal infiltration technique (needle inserted into the mucobuccal fold at ∼45° to bone, small volume deposited upon insertion, needle advanced until bone contact, remaining anesthetic delivered slowly over ∼60 s). All children wore protective eyewear, and in Group B the spray was applied out of the child's line of sight to help preserve blinding. Prior to the intraoral application of either topical anesthetic, each child was prepared with a Tell–Show–Do approach (briefly demonstrating the sensation on the child's hand) to reduce anxiety and ensure they understood the procedure. No other sedatives or analgesics were used.

### 2.8 Outcomes and assessment timepoints

The primary outcome of this study was physiological stress, which was measured by heart rate (HR) in beats per minute (bpm) using a pediatric finger pulse oximeter (Nellcor™ PM10N, Medtronic, Minneapolis, MN, USA). HR readings were taken at two distinct timepoints: “Before” (a baseline measurement obtained 30 s prior to the application of the topical anesthetic, while the child was seated calmly) and “After” (recorded immediately upon completion of the local anesthetic needle insertion and solution deposition).

The secondary outcomes included the following:

1. Self-Reported Pain: This was assessed using the Faces Pain Scale (FPS) (32). The FPS was presented to the child prior to any intervention (Before) to measure anticipatory anxiety and pain expectation and immediately following the injection (After) to evaluate the pain experienced. Scores were captured on a scale ranging from 0 (no pain) to 8 (worst pain), with discrete integer values (0, 1, 2, 3, 4, 5, 6, 8) selected by the child from depicted faces.
2. Behavioral Response: This was evaluated using the Sound-Eye-Motor (SEM) scale. Each domain (Sound, Eye, Motor) was rated from 1 (indicating comfort/no distress) to 4 (indicating severe distress), yielding a composite score ranging from 1 to 4, calculated as the average across the domains. SEM assessments were conducted “Before” (during the application of the topical anesthetic agent) and “After” (during the needle insertion and injection of the local anesthetic solution).

### 2.9 Data collection and management

All patient data were collected using standardized forms during the treatment visit. The study's time frame for data collection was August–December 2023. Pain response measurements (HR readings, SEM scores, and FPS scores) were recorded immediately after each injection. After data collection, records were de-identified by assigning codes to participants and removing personal information. The compiled dataset was double-checked for completeness and accuracy, then entered into a computer database by the primary investigator. Participant confidentiality was maintained throughout by storing data securely and using anonymized codes in analysis.

### 2.10 Statistical analysis

Data were analyzed using IBM SPSS Statistics (Version 29). The Shapiro–Wilk test was used to assess normality of data distributions. (Table 2) As most outcome variables significantly deviated from normality (*p* < 0.05), non-parametric tests were employed throughout.

Baseline demographic characteristics were compared between groups using the Mann–Whitney *U* test (age) and the Chi-square test (gender). The primary between-group comparison was conducted on the change scores (After minus Before) for each outcome using the Mann–Whitney *U* test. Effect estimates were reported as Hodges–Lehmann median differences with 95% confidence intervals. Additionally, bootstrap 95% confidence intervals (10,000 resamples) on the mean difference in change scores were calculated. Within-group pre-post changes were assessed using the Wilcoxon signed-rank test. A two-sided *p*-value of <0.05 was considered statistically significant.

Post-hoc exploratory subgroup analyses by age group (7–8 years vs. 9–10 years) and gender were conducted and are explicitly labeled as exploratory.

## 3 Results

### 3.1 Participant flow and baseline characteristics

Eighty children were assessed for eligibility. Twenty-two were excluded (18 did not meet inclusion criteria due to Frankl I/II behavior; 4 declined to participate). Fifty-eight were randomized (29 to each group). One participant in Group B (EC) was excluded post-randomization due to a protocol violation (incorrect LA technique), leaving 57 participants in the final analysis (Group A: *n* = 29; Group B: *n* = 28).

**Figure 3:**
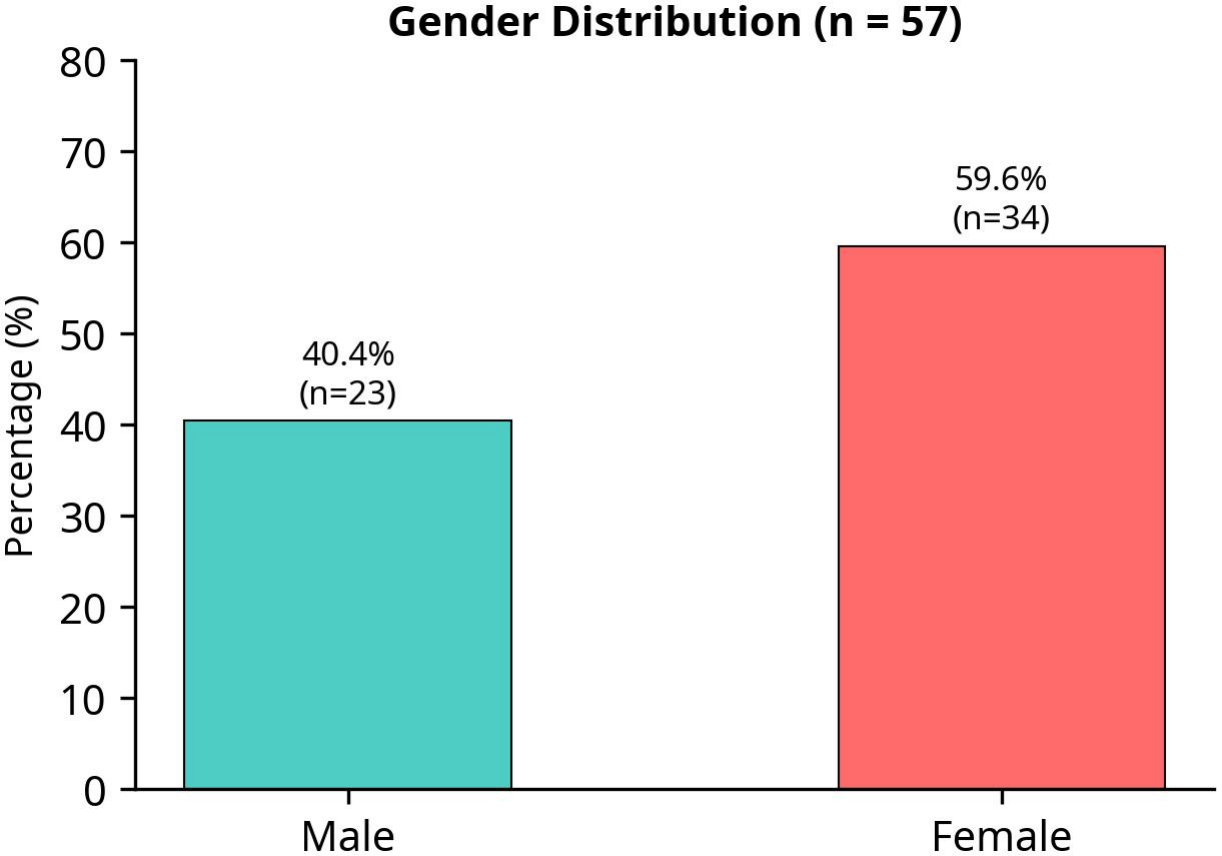
Gender distribution of participants by treatment group.

The two groups were well balanced at baseline. The median age was 9 years (IQR: 8–10) in both groups (Mann–Whitney *U p* = 0.887). Gender distribution was comparable: males comprised 41.4% of Group A and 39.3% of Group B (*χ*^2^ *p* = 0.543) (Table 1) (Figure 3).

**Table 1:** Baseline demographic characteristics by randomized group.

Characteristic	Group A (BC) *n* = 29	Group B (EC) *n* = 28	*p*-value
Age, median [IQR]	9 [8, 10]	9 [8, 10]	0.887*
Age, mean (SD)	8.83 (1.20)	8.79 (1.20)	
Gender—male, *n* (%)	12 (41.4%)	11 (39.3%)	0.543**
Gender—female, *n* (%)	17 (58.6%)	17 (60.7%)	

* Mann–Whitney *U* test.

** Chi-square test.

The Shapiro–Wilk test indicated that most study variables were not normally distributed (*p* < 0.05). Heart rate before the intervention deviated significantly from normality in both groups, whereas heart rate after the intervention was approximately normally distributed (Group A: *p* = 0.053; Group B: *p* = 0.297). All pain assessment measures (Visual Analog Scale, Facial Pain Scale, and Sound–Eye–Motor scores) before and after the intervention showed significant departures from normality in both groups (all *p* < 0.05). Therefore, non-parametric statistical methods are appropriate for analyzing pain-related outcomes, while parametric methods may be considered for post-intervention heart rate if other assumptions are satisfied (Table 2).

**Table 2:** Tests of normality (Shapiro–Wilk).

Variable	Group	Statistic	df	*p*-value
HR (bpm) before	A (BC)	0.914	29	0.022
HR (bpm) before	B (EC)	0.922	28	0.040
HR (bpm) after	A (BC)	0.929	29	0.053
HR (bpm) after	B (EC)	0.957	28	0.297
FPS before	A (BC)	0.643	29	<0.001
FPS before	B (EC)	0.687	28	<0.001
FPS after	A (BC)	0.867	29	0.002
FPS after	B (EC)	0.835	28	<0.001
SEM before	A (BC)	0.424	29	<0.001
SEM before	B (EC)	0.445	28	<0.001
SEM after	A (BC)	0.462	29	<0.001
SEM after	B (EC)	0.560	28	<0.001

### 3.2 Primary outcome: heart rate

**Figure 4:**
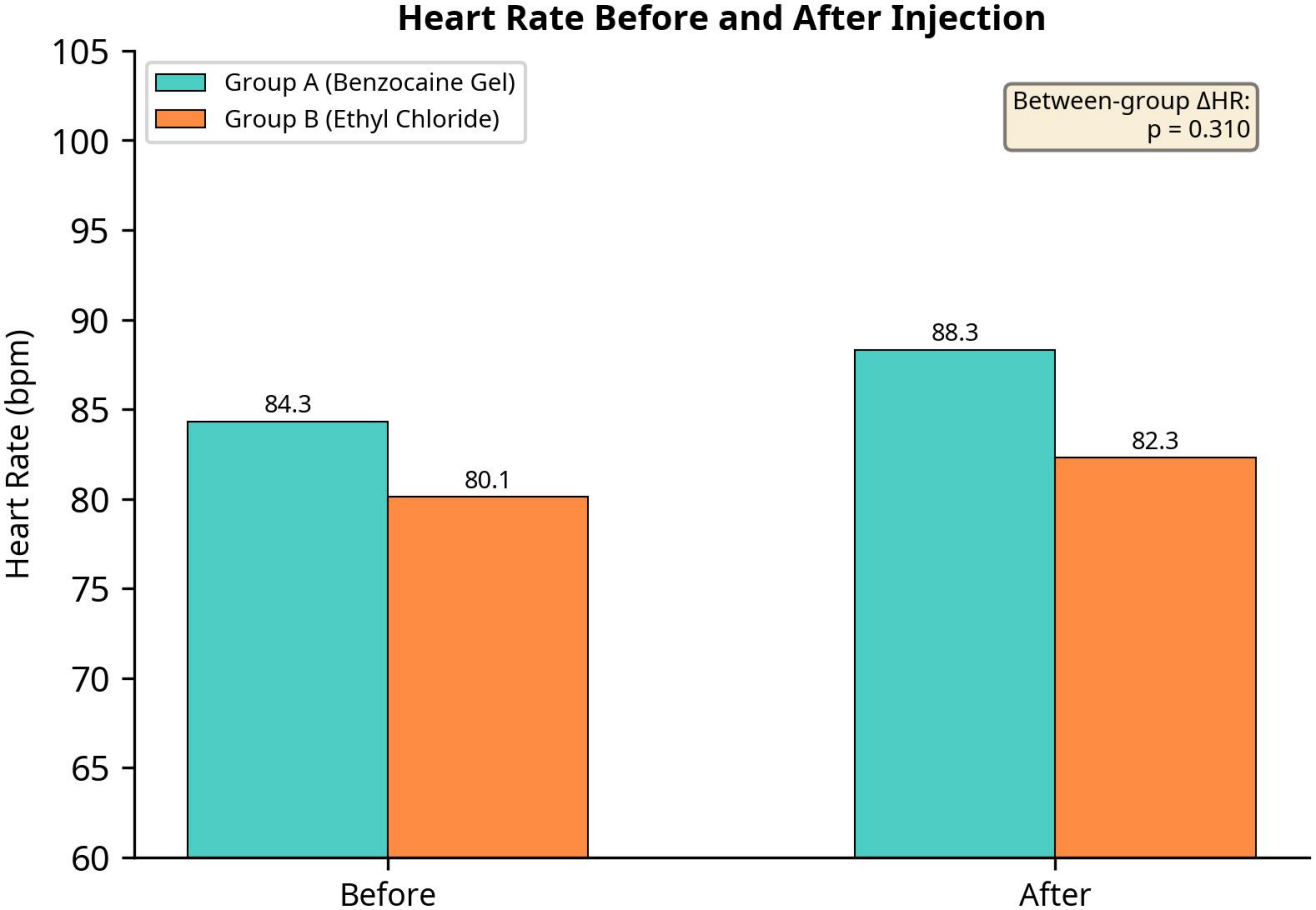
Mean heart rate (HR)/bpm before and after local anesthetic injection by treatment group.

Both groups exhibited a slight increase in heart rate from baseline to post-injection. In Group A (BC), the mean HR increased from 84.3 bpm (SD 15.1) to 88.3 bpm (SD 16.9), representing a mean change of +4.03 bpm (SD 7.37; 95% CI: 1.23–6.84). In Group B (EC), the mean HR increased from 80.1 bpm (SD 12.7) to 82.3 bpm (SD 15.0), representing a mean change of +2.14 bpm (SD 6.11; 95% CI: −0.22 to 4.51) (Table 3, Figure 4).

**Table 3:** Descriptive statistics for all outcomes stratified by group.

Outcome	Group A (BC) *n* = 29 mean (SD)	Group A (BC) median [Q1, Q3]	Group B (EC) *n* = 28 mean (SD)	Group B (EC) median [Q1, Q3]	*p*-value*
HR before (bpm)	84.3 (15.1)	85 [73, 90.5]	80.1 (12.7)	80 [71, 87.8]	0.190
HR after (bpm)	88.3 (16.9)	87 [79.5, 97]	82.3 (15.0)	82 [69.3, 92]	0.199
FPS before	1.03 (1.74)	0 [0, 2]	0.68 (1.02)	0 [0, 1.8]	0.589
FPS after	2.34 (2.33)	2 [0, 4]	1.93 (2.02)	1.5 [0, 4]	0.487
SEM before	1.17 (0.47)	1 [1, 1]	1.25 (0.65)	1 [1, 1]	0.678
SEM after	1.28 (0.70)	1 [1, 1]	1.36 (0.73)	1 [1, 1.8]	0.508

* Mann–Whitney *U* test (between-group comparison at each timepoint).

The between-group comparison of HR change scores revealed no statistically significant difference (Mann–Whitney *U* = 469.5, *p* = 0.310) (Table 4) The Hodges–Lehmann median difference was 2.00 bpm (BC higher), and the bootstrap 95% CI on the mean difference was −1.53 to 5.35 bpm. As this confidence interval includes zero, the null hypothesis of no difference cannot be rejected.

**Table 4:** Between-group comparison of change scores (primary analysis).

Outcome (change = after—before)	Group A (BC) mean (SD)	Group A (BC) 95% CI	Group B (EC) mean (SD)	Group B (EC) 95% CI	Mann–Whitney *p*-value	HL median diff.	Bootstrap 95% CI of mean diff.
Heart rate (bpm)	+4.03 (7.37)	1.23, 6.84	+2.14 (6.11)	−0.22, 4.51	0.310	2.00	−1.53, 5.35
FPS	+1.31 (2.24)	0.46, 2.16	+1.25 (1.84)	0.54, 1.96	0.546	0.00	−1.03, 1.08
SEM	+0.10 (0.41)	−0.05, 0.26	+0.11 (0.42)	−0.05, 0.27	0.971	0.00	−0.22, 0.21

HL, Hodges–Lehmann. Bootstrap: 10,000 resamples. All comparisons non-significant (*p* > 0.05).

### 3.3 Secondary outcome: faces pain scale (FPS)

**Figure 5:**
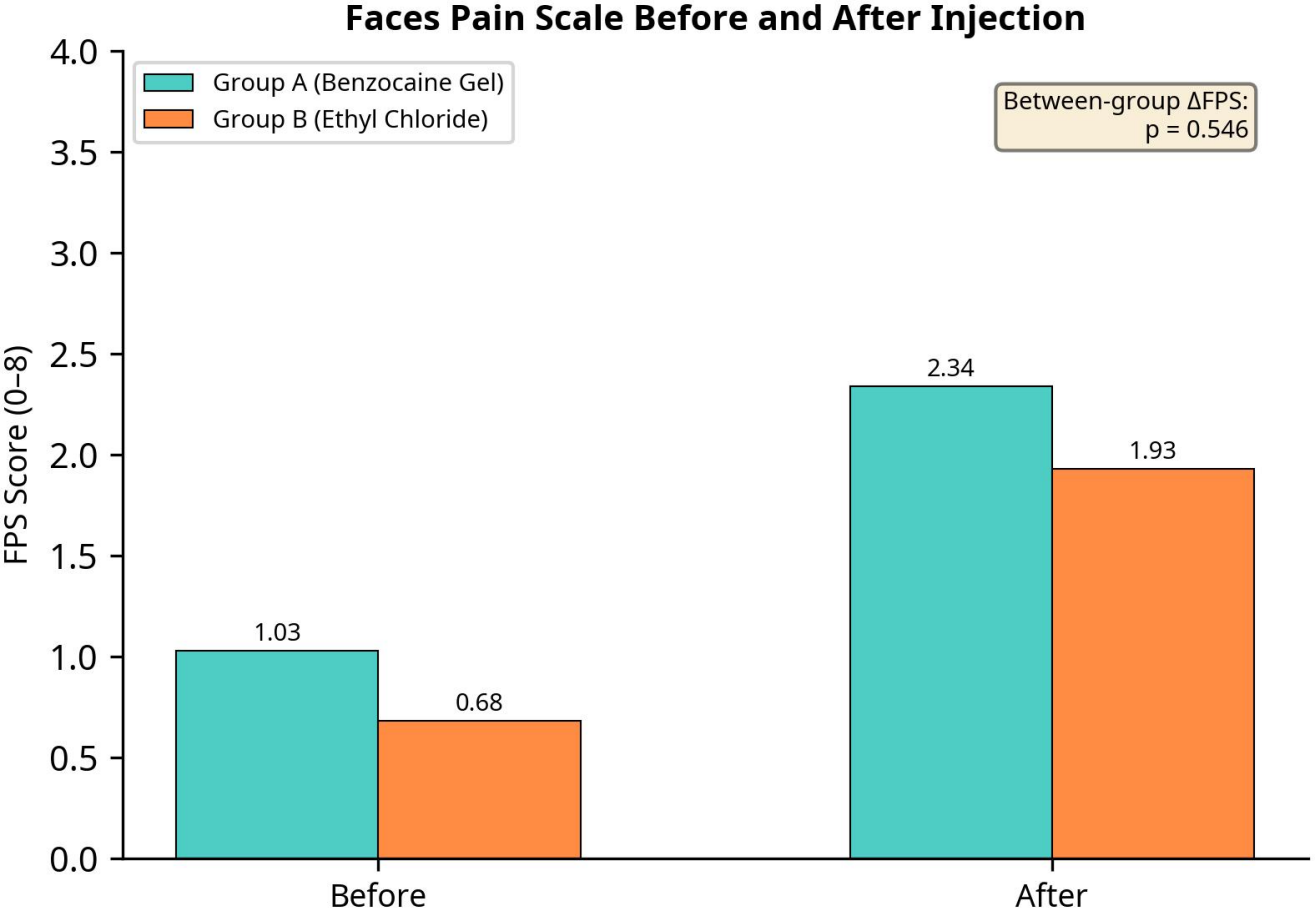
Mean Faces Pain Scale (FPS) scores before and after local anesthetic injection by treatment group.

Self-reported pain increased from baseline in both groups following the injection. In Group A (BC), the mean FPS increased from 1.03 (SD 1.74) to 2.34 (SD 2.33), a mean change of +1.31 (SD 2.24). In Group B (EC), the mean FPS increased from 0.68 (SD 1.02) to 1.93 (SD 2.02), a mean change of +1.25 (SD 1.84) (Figure 5).

The between-group comparison showed no statistically significant difference in FPS change scores (Mann–Whitney *U* = 442.5, *p* = 0.546). The Hodges–Lehmann median difference was 0.00, and the bootstrap 95% CI on the mean difference was −1.03 to 1.08.

### 3.4 Secondary outcome: sound-eye-motor (SEM) scale

**Figure 6:**
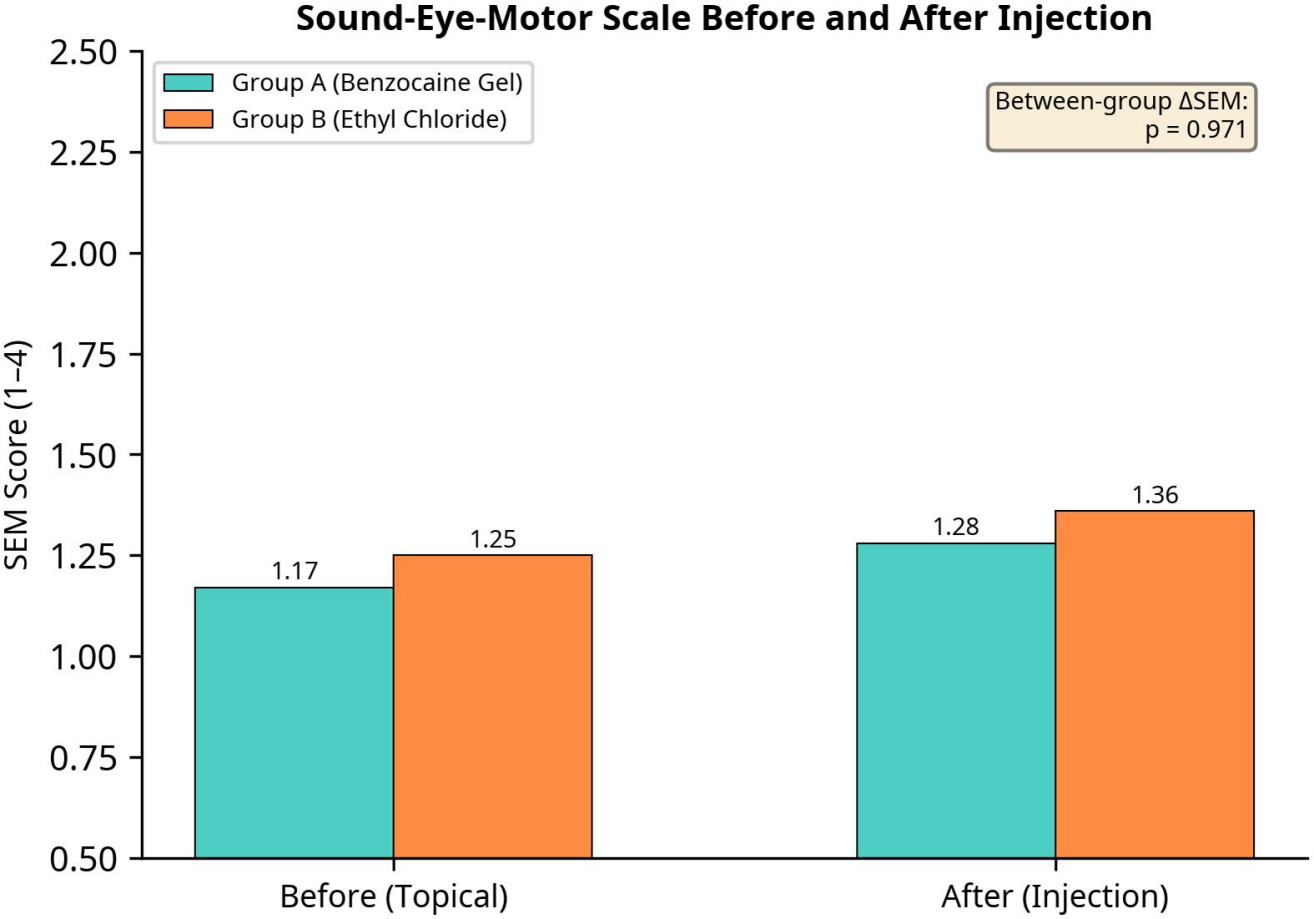
Mean sound, eye, motor (SEM) scores before and after local anesthetic injection by treatment group.

Behavioral responses showed minimal change from the topical application phase to the injection phase in both groups. In Group A (BC), the mean SEM score changed from 1.17 (SD 0.47) to 1.28 (SD 0.70), a mean change of +0.10 (SD 0.41). In Group B (EC), the mean SEM score changed from 1.25 (SD 0.65) to 1.36 (SD 0.73), a mean change of +0.11 (SD 0.42) (Figure 6).

The between-group comparison revealed no statistically significant difference (Mann–Whitney *U* = 405.0, *p* = 0.971). The Hodges–Lehmann median difference was 0.00, and the bootstrap 95% CI on the mean difference was −0.22 to 0.21.

### 3.5 *Post-hoc* exploratory subgroup analyses

Exploratory subgroup analyses stratified by age group (7–8 years, *n* = 22 vs. 9–10 years, *n* = 35) and gender (male, *n* = 23 vs. female, *n* = 34) revealed no statistically significant interactions with the treatment group for any outcome (all *p* > 0.05). These analyses were not pre-specified and should be interpreted with caution given the small subgroup sizes.

### 3.6 Adverse events

No adverse events were observed during the study procedures. Specifically, no cases of visible mucosal frostbite, blanching, or tissue damage were noted following EC application. However, it should be noted that no formal post-operative mucosal follow-up protocol was implemented; therefore, delayed adverse effects cannot be excluded.

## 4 Discussion

This assessor-blind, parallel-group, superiority randomized controlled trial was conducted to assess the efficacy of indirect ethyl chloride (EC) spray application via a cotton pellet in providing pain reduction and physiological stress attenuation during pediatric intra-oral infiltration injections, in comparison to conventional 20% benzocaine (BC) gel. The results of the trial did not indicate a statistically significant difference between the two agents across any measured outcomes. Both EC and BC demonstrated comparable effects in relation to physiological measures (heart rate), self-reported pain assessments (Faces Pain Scale), and behavioral responses (Sound-Eye-Motor measures).

The primary outcome, heart rate change, was selected as an objective physiological marker of acute procedural stress (1, 26). While both groups experienced a slight elevation in HR following the injection, the between-group difference in the magnitude of this increase (+4.0 bpm for BC vs. +2.1 bpm for EC) was neither statistically significant (*p* = 0.310) nor clinically meaningful. The bootstrap 95% confidence interval for the mean difference (−1.53 to 5.35 bpm) spans zero, confirming that neither agent demonstrated a clear advantage in suppressing the autonomic stress response. These findings are consistent with Vellanki et al., who found that while EC provided superior analgesia for pediatric intravenous cannulation compared to lignocaine spray, the physiological stress markers were comparable between groups (11).

The physiological mechanism underlying cryoanesthesia using EC relies on the rapid cooling of the mucosal surface, which slows or blocks the conduction of nerve impulses in local nociceptors and simultaneously activates cold receptors, thereby modulating pain perception via the gate control theory (5, 7). When ethyl chloride evaporates from the cotton pellet surface, it produces a rapid temperature drop to approximately −20 °C, sufficient to induce transient numbness of the superficial nerve endings within 15–30 s. This mechanism is distinct from the pharmacological action of benzocaine, which blocks sodium channels in nerve membranes, requiring 60–120 s for clinical onset (3, 10). Despite these differing mechanisms, both methods achieved comparable pain attenuation in the present trial, suggesting that the clinical endpoint—reduced nociceptive transmission during needle penetration- can be reached through either pathway with equivalent efficacy.

Our findings align with previous research on the use of cooling techniques to reduce injection pain in dentistry (15, 16, 27, 28). Harbert (17) demonstrated that topical ice application could reduce pain during palatal anesthesia, while Hameed et al. (29) showed that pre-cooling the injection site with a tetrafluoroethane refrigerant spray significantly decreased pain perception in children aged 8–12 years. Lakshmanan et al. (15) reported that cryotherapy was effective in attenuating both pain and anxiety associated with dental injections, and Hindocha et al. (16) found ice to be comparable to 5% lidocaine gel for topical mucosal anesthesia. While several of these earlier studies reported superiority of cooling methods over no treatment, our trial—which compared EC against an active gold-standard comparator (20% benzocaine)—demonstrates therapeutic equivalence rather than superiority. The rapid and potent analgesic efficacy of EC has also been recently corroborated in broader pediatric settings; Vellanki et al. (11) demonstrated that EC spray provided significantly superior dermal analgesia and faster onset compared to lignocaine spray for pediatric intravenous cannulation, with no reported adverse effects. The present study's multi-modal assessment (heart rate, SEM, FPS) provides a robust evaluation that corroborates the subjective relief with objective physiological data, strengthening the case for cryoanesthesia in pediatric dental practice.

Self-reported pain (FPS) similarly showed no between-group difference (*p* = 0.546). Both groups reported mild-to-moderate increases in pain following the injection (BC: +1.31; EC: +1.25 on the FPS), with the 95% CI (−1.03 to 1.08) firmly spanning zero. This finding aligns with Ninawe and Anija, who reported that ice application and benzocaine gel produced statistically comparable FPS scores in children receiving local anesthesia (19). The Sanglard et al. scoping review of 52 pediatric trials also concluded that cooling-based methods and pharmacological topical agents demonstrate broadly similar efficacy profiles (18).

The SEM behavioral scores showed negligible change in both groups (BC: +0.10; EC: +0.11; *p* = 0.971). This near-zero change must be interpreted in the context of the study's inclusion criteria. By restricting enrollment to children with Frankl III or IV behavior ratings, the study pre-selected a highly cooperative cohort who were already at or near the floor of the SEM scale (baseline means of 1.17 and 1.25 on a 1–4 scale). This floor effect inherently limits the ability to detect behavioral improvements from any topical anesthetic intervention. Future studies should consider including children with a broader range of baseline anxiety levels (including Frankl II) to better capture the potential behavioral benefits of rapid-onset cryoanesthesia.

Our exploratory subgroup analyses additionally hint at potential differences in pain experiences by age and gender. While not statistically significant, older children (9–10 years) exhibited slightly smaller HR increases and possibly better coping (lower SEM scores) than younger children (7–8 years), consistent with the idea that as children mature, they may manage injection stress more effectively (30). Also, in line with previous research, female children in our sample showed a tendency toward higher anxiety or behavioral discomfort indicators (SEM), whereas male children reported slightly higher pain intensity (FPS). These subtle trends mirror known patterns in pediatric pain studies, where older children and boys sometimes report pain differently than younger children and girls (31). However, our sample size was not powered to detect small subgroup differences, so these observations require cautious interpretation and further exploration in larger cohorts.

Beyond the pharmacological equivalence demonstrated in this trial, the indirect ethyl chloride technique offers several practical and clinical advantages over conventional benzocaine gel that merit clinical consideration. Conventional topical anesthetics such as benzocaine require 1–3 min of mucosal contact before achieving adequate surface anesthesia (3), during which time the child must remain still with the agent in place—a challenging expectation in anxious pediatric patients. In contrast, the EC cotton pellet achieved its cryoanesthetic effect within 15–30 s of application, substantially reducing the chair-side waiting period and minimizing the window for behavioral non-compliance.

Furthermore, benzocaine gel produces collateral soft-tissue numbness that extends beyond the injection site and persists for 15–30 min after the procedure (3, 4). This residual numbness of the lip, cheek, or tongue is particularly problematic in young children, who may inadvertently bite or chew the anesthetized tissues, leading to traumatic ulceration—a well-recognized post-operative complication in pediatric dentistry (10). The EC technique, by contrast, produces only transient, highly localized numbness at the precise application site that dissipates within seconds of pellet removal, effectively eliminating the risk of self-inflicted soft-tissue injury. Additional drawbacks of benzocaine include its unpleasant bitter taste, which can provoke gagging or non- compliance in children, and its higher material cost relative to a single-use cotton pellet sprayed with EC (8, 14).

Importantly, no adverse effects were observed with either method in this trial. Although direct spraying of ethyl chloride onto oral mucosa carries a theoretical risk of localized frostbite, our indirect application protocol—spraying onto a cotton pellet until frost formation, then applying the pellet to the buccal mucosa for a controlled 30 s with protective eyewear effectively prevented such complications. This approach also allowed children to become comfortable with the cold sensation through a brief demonstration on their hand before intraoral application, which may have contributed to the low behavioral distress scores observed in the EC group.

Both the EC and benzocaine methods permitted the necessary dental procedures to be completed successfully without major behavioral disruption; however, the favorable safety profile, rapid onset, absence of residual numbness, low cost, and simple application technique collectively position the indirect EC spray method as a viable alternative to benzocaine 20% gel for routine pre-injection topical anesthesia in cooperative pediatric dental patients.

Key strengths of this study include its randomized controlled design with blinded outcome assessment, which strengthens the validity of the findings, and the use of multiple pain measures (heart rate, behavior, and self-report) to capture different dimensions of the child's pain experience. The novel cryoanesthesia technique was standardized and simple, making the findings readily translated to clinical practice. However, the trial was conducted at a single center with a moderate sample size, which may limit the generalizability of the results. The study population was restricted to cooperative children aged 7–10 years undergoing buccal infiltrations; results might differ for other ages, less cooperative children, or other injection types (e.g., palatal injections).

### 4.1 Limitations

Several limitations should be acknowledged. First, the inability to blind the operator and the child participants is an inherent constraint of comparing a cryogenic agent (with a distinct cold sensation) to a room-temperature gel. While the strict assessor-blind protocol mitigated observer bias for the SEM outcome, the subjective FPS self-report may have been influenced by the child's awareness of the intervention received. Second, the study did not include a negative control group (no topical anesthesia). This was a deliberate ethical decision, as withholding pre-injection topical anesthesia from pediatric patients is considered below the standard of care. Third, the sample size was powered to detect a large effect (*d* = 0.82); smaller but potentially clinically relevant differences may have been missed. Fourth, no formal post-operative mucosal follow-up was conducted. While no acute adverse events were observed, the long-term mucosal safety of repeated indirect EC application requires further investigation. Fifth, the Kryogen R&S product is labeled for use on intact skin; its off-label application to oral mucosa via the indirect cotton-pellet technique requires further safety validation.

## 5 Conclusion

This superiority trial did not demonstrate a statistically significant difference between indirect ethyl chloride spray application and 20% benzocaine gel for any measured outcome, physiological stress (heart rate), self-reported pain (Faces Pain Scale), or behavioral response (Sound-Eye-Motor scale), during intra-oral local anesthetic injections in cooperative children aged 7–10 years. Both topical anesthetic agents produced comparable, clinically acceptable outcomes.

Given its rapid onset (30 s vs. 2–3 min for benzocaine) and the absence of observed acute adverse events, ethyl chloride applied via a cotton pellet may represent a time-efficient alternative to benzocaine gel, though it does not offer superior efficacy. Larger, multi-center trials with broader behavioral inclusion criteria and formal mucosal safety follow-up are warranted.

## Data Availability

The original contributions presented in the study are included in the article/Supplementary Material, further inquiries can be directed to the corresponding author.
